# Contribution of time of day and the circadian clock to the heat stress responsive transcriptome in *Arabidopsis*

**DOI:** 10.1038/s41598-019-41234-w

**Published:** 2019-03-18

**Authors:** Emily J. Blair, Titouan Bonnot, Maureen Hummel, Erika Hay, Jill M. Marzolino, Ivan A. Quijada, Dawn H. Nagel

**Affiliations:** Department of Botany and Plant Sciences, University of California, Riverside, Riverside California, USA

## Abstract

In *Arabidopsis*, a large subset of heat responsive genes exhibits diurnal or circadian oscillations. However, to what extent the dimension of time and/or the circadian clock contribute to heat stress responses remains largely unknown. To determine the direct contribution of time of day and/or the clock to differential heat stress responses, we probed wild-type and mutants of the circadian clock genes *CCA1*, *LHY*, *PRR7*, and *PRR9* following exposure to heat (37 °C) and moderate cold (10 °C) in the early morning (ZT1) and afternoon (ZT6). Thousands of genes were differentially expressed in response to temperature, time of day, and/or the clock mutation. Approximately 30% more genes were differentially expressed in the afternoon compared to the morning, and heat stress significantly perturbed the transcriptome. Of the DEGs (~3000) specifically responsive to heat stress, ~70% showed time of day (ZT1 or ZT6) occurrence of the transcriptional response. For the DEGs (~1400) that are shared between ZT1 and ZT6, we observed changes to the magnitude of the transcriptional response. In addition, ~2% of all DEGs showed differential responses to temperature stress in the clock mutants. The findings in this study highlight a significant role for time of day in the heat stress responsive transcriptome, and the clock through *CCA1* and *LHY*, appears to have a more profound role than *PRR7* and *PRR9* in modulating heat stress responses during the day. Our results emphasize the importance of considering the dimension of time in studies on abiotic stress responses in *Arabidopsis*.

## Introduction

The clock enables organisms to synchronize their metabolism, physiology, and development to predictable daily and seasonal environmental changes conferring enhanced fitness and growth vigor in the plants^1–4^. Time of day information is gathered through key inputs such as light, temperature, and metabolite levels^5–9^. Underlying the clock network are multiple feedback loops, with interconnected components that interact both negatively and positively to influence a wide range of cellular and biological processes^1–3,10–15^. At the core of the oscillator, two closely related Myb domain transcription factors (TFs), CIRCADIAN CLOCK ASSOCIATED1 (CCA1) and LATE ELONGATED HYPOCOTYL (LHY), both expressed in the morning, negatively regulate the expression of the evening phased clock gene *TIMING OF CAB EXPRESSION1 (TOC1*)^16–19^. TOC1 in turn completes the loop by regulating *CCA1* and *LHY* expression in the late evening^16–19^. Besides this core feedback loop, CCA1 and LHY regulate the expression of *PSEUDO RESPONSE REGULATOR 7 (PRR7)* and *PRR9*, which are expressed during the day as part of a morning regulatory loop^20–22^. These four components (*CCA1*, *LHY*, *PRR7* and *PRR9*), along with a few other evening expressed clock genes, are important for maintaining a relatively constant period (~24 h) within a range of growth permissive temperatures (~12–30 °C)^23–25^.

Both temperature and the clock control many aspects of plant growth and fitness through extensive regulation of gene expression^4,26–28^. The clock also directly influences key crop traits while high temperature stress can alter crop productivity^29,30^. Based on transcriptome experiments, up to 50% of the genes responsive to heat, cold, salinity, osmoticum, or water deprivation show circadian rhythmicity in *Arabidopsis*^31–33^. Rhythmic expression of abiotic stress-responsive genes is also observed in soybean and barley^34,35^.

Mechanistically, the clock is able to regulate the expression of these stress responsive genes by controlling the magnitude or occurrence of the response based on time of day, a process referred to as gating^32,36–40^. The relevance of time of day transcriptomic changes for a given stress response such as drought or cold has been comprehensively examined and continues to emerge^37,38,40–42^. For example, cold induction of the *C-REPEAT BINDING FACTORS* (*CBFs;* also known as *DEHYDRATION-RESPONSIVE ELEMENT-BINDING (DREB)* TF transcript levels in response to cold (4 °C) is higher in the early morning (4 h after dawn) versus evening (16 h after dawn), and this response is altered in the *CCA1 (cca1-11)* and *LHY (lhy-21)* clock mutants^43–45^.

Numerous transcriptomic studies following various degrees of high temperature stress have been reported^46–54^. However, most of these studies lack information on the dimension of time, suggesting that some heat stress responsive genes might be overlooked. A recent targeted study suggests a role for the evening expressed clock components *TOC1* and *PRR5* in gating the molecular responses of select genes to warm temperature (high ambient temperature)^55^. However, a global understanding of the contribution of time of day and/or the clock to the high temperature responsive transcriptome during the day period when plants are exposed to maximum heat stress and likely primed for high temperature remains incomplete^56^.

Therefore, to determine to what extent time of day and the circadian clock contribute to differential transcriptional responses under heat stress in *Arabidopsis*, we assayed for transcriptomic changes under temperature stress in the early morning (ZT1) and the early afternoon (ZT6), times when temperature stress responsive genes and the morning and day expressed clock genes also exhibit peak expression^56^. From the thousands of genes that were differentially expressed in response to heat treatment and time of day (ZT1 vs ZT6), ~33% and ~38% are specific to either ZT1 or ZT6, respectively, while ~30% are shared between both time points. In all three categories, the majority (>50%) of the DEGs were upregulated in response to heat stress, and the response is more evident in the early afternoon relative to the early morning. In addition, among all of the DEGs, 2% showed differential expression in response to temperature stress in the clock mutants and also when compared to wild-type. Our analyses have revealed that during the day when plants are exposed to maximum high temperatures, time of day plays an extensive and important role in modulating the heat stress responsive transcriptome, and this sensitivity is more evident in the early afternoon. In addition, the clock through the morning expressed clock genes *CCA1* and *LHY*, also modulates the heat stress responses preferentially in the early morning. In summary, this analysis provides the first global analysis on the contribution of time of day and/or the clock to heat stress responses in *Arabidopsis*.

## Results and Discussion

### Time of day specific transcriptome changes in response to temperature stress

**Figure 1:**
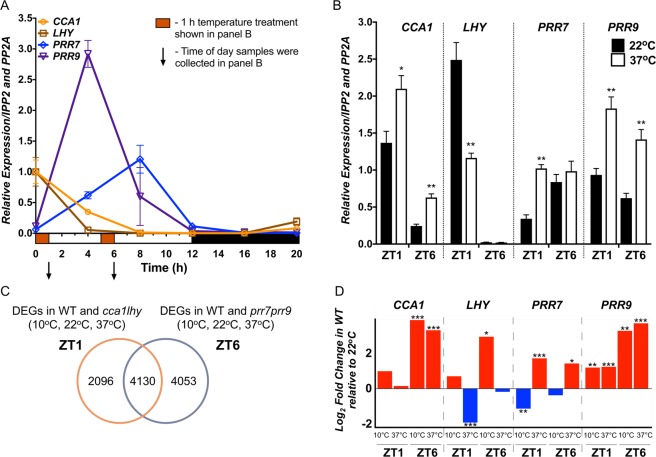
Targeted and global gene expression in response to temperature stress at specific times of the day. (**A**) Expression profile of *CCA1*, *LHY*, *PRR7* and *PRR9* in WT plants grown in 12 h Light:12 h Dark (LD) cycles for 12 d by qRT-PCR to demonstrate the time of peak expression. Hashed box indicates when plants were transferred to 10 °C or 37 °C, and down arrow when samples were collected following temperature treatment, for the results shown in panel B. X-axis, time in hours (h) and Y-axis, relative expression. (**B**) Changes in transcript abundance of *CCA1*, *LHY*, *PRR7*, and *PRR9* following 1 h heat stress treatment (37 °C) as indicated in panel A. mRNA levels were normalized to *IPP2* and *PP2A* expression (mean values ± SD, n = 3, three independent experiments). **P ≤ 0.01; *P ≤ 0.05, unpaired student t-test. X-axis, time of day samples were collected and Y-axis, relative expression. (**C**) Venn diagram depicting the overlapping DEGs between the *cca1lhy* compared to WT at all temperatures (left circle) and the *prr7prr9* compared to WT at all temperature (right circle) datasets. (**D**) Log_2_ Fold Change (LFC) for *CCA1*, *LHY*, *PRR7*, and *PRR9* in WT at 10 °C or 37 °C compared to 22 °C for ZT1 and ZT6 from our RNA-seq data. ***FDR ≤ 0.001; **FDR ≤ 0.01; *FDR ≤ 0.05.

To first determine how the transcriptional response of clock genes to heat stress is altered, we examined changes in transcript abundance of *CCA1*, *LHY*, *PRR7*, and *PRR9*, clock genes that show peak expression throughout the day period (Fig. 1A). Wild-type (WT) *Arabidopsis* seedlings were exposed to heat stress at two times of day, early morning and early afternoon (indicated in Fig. 1A). These time-points were selected because they relatively correspond to the time of day when genes that are upregulated by heat or downregulated by cold show peak expression (dawn) and when the maximum heat stress responses occur^56^. We performed quantitative real-time PCR (qRT-PCR) on samples collected from seedlings grown in 12 h light/12 h dark cycles (LD) and constant temperature (22 °C) for twelve days, then exposed for 1 hour to 37 °C on day thirteen at dawn or early afternoon (Fig. 1A,B). We observe enhanced expression of *CCA1*, *PRR7*, and *PRR9* and a reduction in *LHY* expression under heat stress, confirming that these clock components are responsive to high temperature stress, and therefore, are likely to affect the transcriptional response of their downstream targets (Fig. 1B). This is consistent with a recent transcriptomic report showing that these clock genes are similarly differentially expressed even after 30 mins of heat treatment (37 °C)^57^.

To assess the global gene expression dynamics between time of day, the clock, and heat stress responses, we performed RNA-sequencing (RNA-seq) on WT and mutant *Arabidopsis* seedlings of *CCA1*, *LHY*, *PRR7*, and *PRR9*, using a similar heat treatment (1 h at 37 °C) as described above, and included a moderate cold stress treatment (1 h at 10 °C) to distinguish between heat responsive genes and general temperature responsive genes. We selected the double mutant lines for *CCA1* and *LHY* (*cca1-1/lhy-20*) since these two clock genes showed opposing heat responses, and *PRR7* and *PRR9* (*prr7-3/prr9-1*) to cover both the midday and early afternoon times of the day^21,28,58,59^. Twelve day old WT, *cca1-1/lhy-20* (*cca1lhy*) and *prr7-3/prr9-1* (*prr7prr9*) seedlings were exposed for 1 h to 10 °C or 37 °C at dawn (ZT0, at lights ON) or early afternoon (ZT5, after lights ON) and collected at ZT1 or ZT6, respectively^21,28,59,60^. We filtered the resulting datasets for genes with a Log_2_ Fold Change (LFC) > |1| and False Discovery Rate (FDR) <0.05 to be more inclusive and to consider that even small differences in expression levels might have a significant impact on the regulation of some genes (Supplementary Dataset S1 and S2). Based on these criteria, we obtained 6266 and 8183 DEGs that represent both the genotype and temperature condition at ZT1 and ZT6, respectively (Fig. 1C; Supplementary Dataset S3). Most of the known clock genes exhibit differential expression in response to temperature stress (Supplementary Dataset S4)^61^. Consistent with our qRT-PCR results in Fig. 1B, *CCA1*, *PRR7*, and *PRR9* showed upregulation and *LHY* downregulation following heat stress (37 °C) (Fig. 1D). However, based on our FDR cut-off criteria, *CCA1* upregulation at 37 °C is only significant at ZT6, while *LHY* downregulation is significant at ZT1 (Fig. 1D). At 10 °C, *CCA1* and *PRR9* appear to also be upregulated, while *LHY* and *PRR7* exhibit opposite expression, upregulated and downregulated, respectively (Fig. 1D). Overall, more genes were differentially expressed at ZT6 vs ZT1, and although 4130 DEGs are shared between the ZT1 and ZT6 datasets, approximately 50% are specific to each dataset, emphasizing the importance of time of day in transcriptomic analysis when assaying for temperature stress responses (Fig. 1C).

As multiple temperature stress related genome-wide experiments have been performed, we compared our list of DEGs with a selected subset of expression datasets available through Genevestigator along with the most recently published heat and cold RNA-seq experiments at the time of this analysis^42,46,57^. While most of our DEGs were shared with these other experiments, we identified DEGs that are specific to either our cold (2.77%) or heat stress (7.94%) DEGs (Supplementary Fig. S1 and Dataset S3). Because we did not account for differences due to analysis pipeline, treatment duration, plant growth conditions, developmental stage, and all published or unavailable experiments, this comparison is not fully conclusive. Although the upregulation of *CCA1* at 37 °C is not significant in our dataset at ZT1 in WT, it is significantly upregulated in the Albihlal *et al*., 2018 dataset, where the growth conditions, duration of 37 °C treatment, and analysis pipeline differ, and information on the time of day the treatment was applied, is unknown. Similarly, *TOC1* is significantly upregulated at 37 °C in the Albihlal *et al*., 2018 but is not differentially expressed in our WT dataset based on our selection criteria, highlighting the limitations of comparative analysis between multiple available data sources that are derived from different experimental conditions and analysis pipeline, etc. (Supplementary Dataset S3).

### Majority of the DEGs show differential response to heat stress

**Figure 2:**
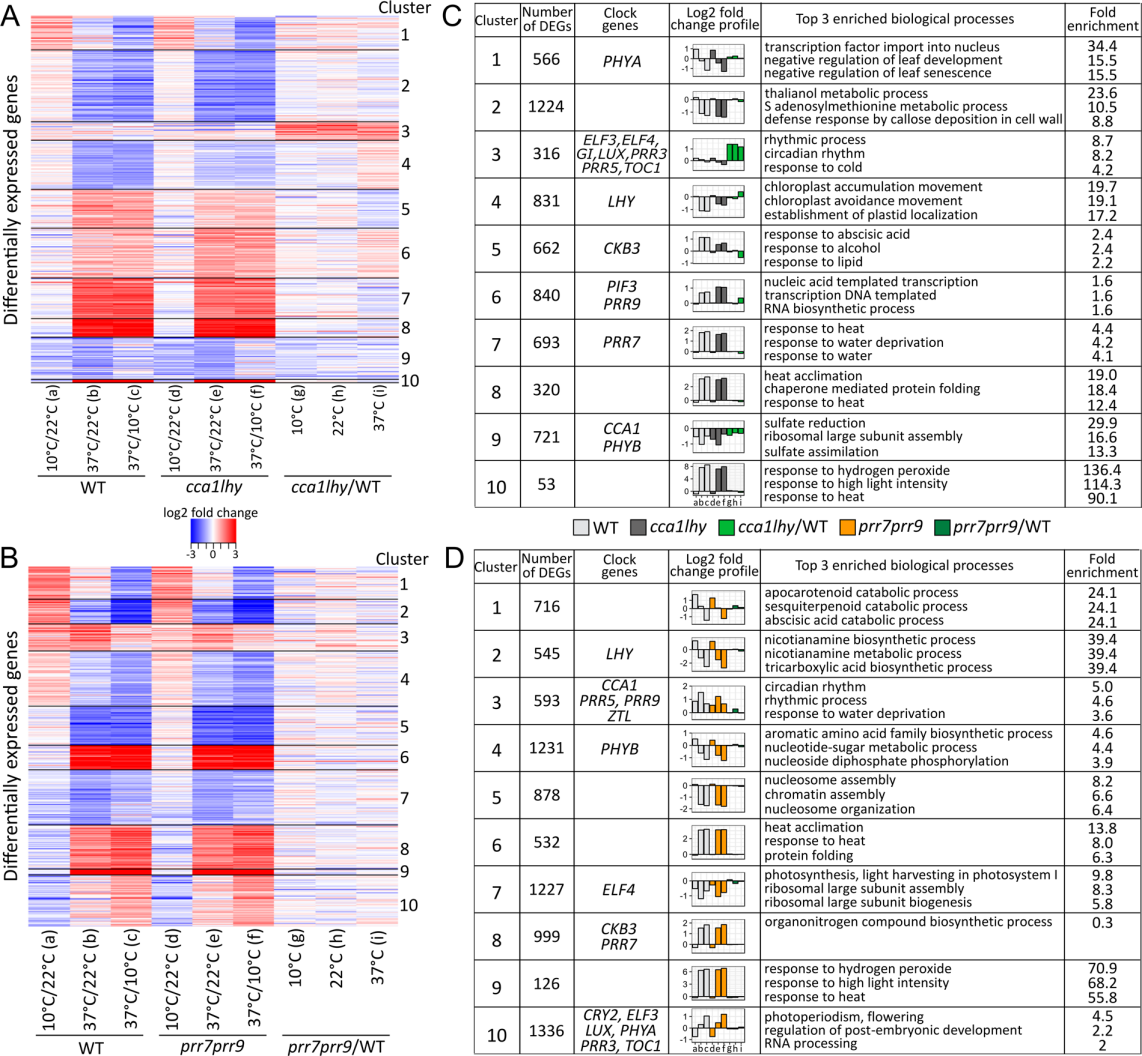
Temperature stress perturbs the transcriptome. (**A**,**B**) Heatmap showing differential expression patterns for DEGs (filtered for LFC > |1| and FDR < 0.05) in *cca1lhy* or *prr7prr9* compared to WT at all temperatures. (**C**,**D**) Summary by cluster of number of genes, clock gene location, general expression pattern measured by LFC, and top 3 enriched biological process gene ontology (GO) terms.

From all of the genotype and condition specific datasets, we first assessed the overall effects of temperature treatment in the context of time of day or the clock mutants on the transcriptome. In both the ZT1 and ZT6 datasets, the treatment at 37 °C highly perturbed the transcriptome compared to 10 °C in the WT and clock mutants (Fig. 2A,B; Supplementary Dataset S5). For example, in the WT, while 3199 (ZT1) and 3359 (ZT6) genes showed differential expression at 37 °C relative to 22 °C, only 256 (ZT1) and 970 (ZT6) genes were differentially expressed at 10 °C compared to 22 °C in WT (Supplementary Fig. S2). Similar numbers of genes were upregulated and downregulated at ZT1 (1771 and 1598) and ZT6 (1970 and 1787) in response to heat stress (37 °C/22 °C; Supplementary Fig. S2). Consistent with this observation, enriched gene ontology (GO) functional categories include processes related to high temperature stress (response to heat and heat acclimation), as indicated in clusters 8 and 10 in the ZT1 dataset and clusters 6 and 9 for ZT6 dataset, and include many of the known *Heat Shock Transcription Factors (HSFs)* and *Heat Shock Proteins (HSPs)* (Fig. 2C,D; Supplementary Dataset S6)^62^. In these clusters (8 and 10 for ZT1; 6 and 9 for ZT6), DEGs were primarily upregulated in response to heat stress. In addition to heat stress related categories, additional enriched categories include response to abscisic acid (ABA), alcohol and lipids, and transcription. DEGs downregulated in response to heat stress at ZT1, in clusters 2, 4, and 9, were mostly enriched in biological processes such as metabolic processes, chloroplast accumulation and movement, and sulfate reduction and assimilation (Fig. 2C and Supplementary Dataset S6). At ZT6, in addition to heat stress related processes, upregulated DEGs were also enriched for circadian rhythms, rhythmic process, and response to water deprivation, while down-regulated DEGs were enriched for metabolic processes, nucleosome organization and assembly, ribosomal large subunit biogenesis/assembly, and photosynthesis (Fig. 2D and Supplementary Dataset S6).

Because the *cca1lhy* mutant exhibits an earlier shift in peak gene expression and the *prr7prr9* mutant a delayed shift in peak gene expression in light dark cycles, direct comparison between WT and mutants could lead to confounding results, specifically false positives, since differential expression at a single time-point could reflect a shift in expression rather than actual differences in peak expression in response to temperature stress between the genotypes^21,63–65^. However, we nevertheless wanted to determine whether the DEGs enriched in some clusters in WT or clock mutants at ZT1 and ZT6 in response to heat stress can be linked to clock function. We observed that only at ZT1, DEGs in cluster 3 that contained either up- or down-regulated genes in WT or *cca1lhy* mutant at 37 °C, showed up-regulation in the *cca1lhy* mutant to WT comparison (Fig. 2A). This cluster was enriched for GO terms such as circadian rhythm, rhythmic process, and response to cold, and most of the evening expressed circadian clock genes grouped within this cluster (Fig. 2C).

### Time of day contributes to differential transcriptional responses under heat stress

**Figure 3:**
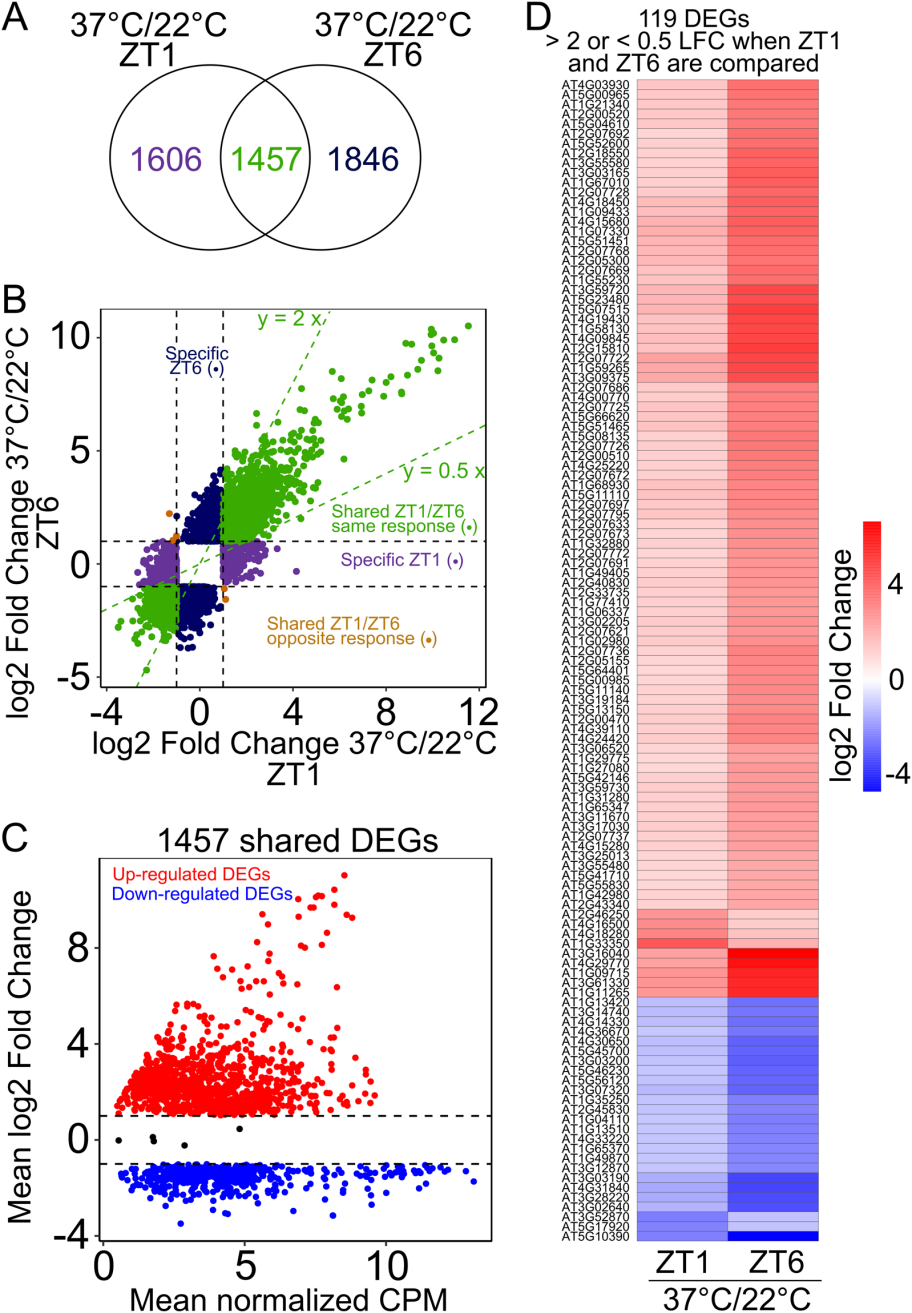
Time of day modulation of heat stress responses. (**A**) Venn diagram representing overlap of genes differentially expressed at ZT1 and ZT6 in response to 37 °C compared to 22 °C. (**B**) Scatter plot comparing Log_2_ Fold Change (LFC) values at ZT1 and ZT6 for the genes differentially expressed in response to 37 °C compared to 22 °C. Genes specific for ZT1 (1606, purple dots), ZT6 (1846, blue dots), and shared between ZT1 and ZT6 (1457, green dots) are plotted. Brown dots represent 5 DEGs that showed opposite expression in ZT1 vs ZT6 (**C**) Scatter plot representing mean LFC and mean normalized counts per million (CPM) reads for the shared (1457) genes upregulated (red dots) or downregulated (blue dots) in response to 37 °C compared to 22 °C. Black dots indicate the 5 DEGs with opposite expression. For each gene, mean of the LFC at ZT1 and ZT6 and the normalized CPM at ZT1 and ZT6 for 22 °C and 37 °C are represented. (**D**) Heatmap showing LFC values at ZT1 and ZT6 for 119 selected heat responsive DEGs with a LFC either ZT1 or ZT6 with a ratio y > 2x or y < 0.5x when these two timepoints were compared.

In nature, the early morning and early afternoon are times of the day when heat responsive genes are most highly expressed. As described above, for both ZT1 and ZT6, a similar number of genes, 3199 (51%) and 3027 (48%) were differentially expressed in response to 37 °C in the WT and in the *cca1lhy* mutant, and 3359 (41%) and 3432 (42%) DEGs in the WT and in the *prr7prr9* mutant, respectively (Supplementary Fig. S2 and Dataset S3). Therefore, we were interested to determine the extent to which the occurrence or magnitude of the transcriptional response to heat stress is modulated by the time of day the stress was applied (ZT1 vs ZT6). For this we compared the ZT1 (3369) and ZT6 (3757) DEGs in response to 37 °C in WT (Supplementary Fig. S2). Approximately 50% are specific to either ZT1 (1742 DEGs) or ZT6 (1902 DEGs), suggesting that the occurrence of the response is specific to certain times of the day (Supplementary Dataset S7). However, because some of these genes also show differential response to moderate cold stress (10 °C), we considered these DEGs as general temperature stress responsive genes and thus excluded them from the heat specific analysis. We obtained 1606 DEGs specific to ZT1 and 1846 DEGs specific to ZT6, where the occurrence of the transcriptional response to heat stress depends on the time of day the treatment was applied (ZT1 vs ZT6; Fig. 3A,B).

In terms of modulation of the response depending on time of day, ~30% (1457) of the DEGs were shared between ZT1 and ZT6, many of which showed time of day specific transcriptional changes (Fig. 3A,B; Supplementary Dataset S7). Most DEGs (~65%) are upregulated in response to 37 °C and enriched for GO biological processes involved with heat stress such as, cellular response to chaperone mediation and unfolded protein, heat acclimation, and hydrogen peroxide, as expected (Fig. 3C and Supplementary Dataset S6). Although a lesser number of genes (~35%) that show differential expression between ZT1 and ZT6 at 37 °C are downregulated, these DEGs are enriched for GO biological processes photosynthesis, response to light, cell wall modification, nucleosome assembly, and metabolic processes (Fig. 3C and Supplementary Dataset S6). Furthermore, ~2/3 (937/1457) of the transcriptional responses exhibit a greater induction (upregulated DEGs) or repression (downregulated DEGs) at ZT6 (Supplementary Dataset S7). We further examined these shared time of day specific DEGs (1457) and found that ~8% (119) show either a LFC ratio >2 or <0.5 between the LFC at ZT1 and ZT6 with a greater LFC at ZT6 (Fig. 3D). These 119 DEGs were not significantly enriched for any specific GO processes. However, several of these genes are characterized as hypothetical proteins of unknown function, and could be interesting candidates for follow-up studies on time of day control of heat stress responses (Fig. 3D). It has recently been shown that alterations to the phase and period of clock and clock controlled genes in response to heat stress, occurs after ~12 h under constant exposure to relatively high temperature (35 °C)^66^. Therefore, we reasoned that differential expression of clock genes and clock output genes in response to acute heat stress at ZT1 and ZT6 after lights ON are most likely due to the time of the day the stress was applied.

Only a few DEGs (5 genes), mostly of unknown function, showed opposing responses between ZT1 and ZT6 at 37 °C (Fig. 3B,C). Of note, in the comparison between ZT1 and ZT6 for the DEGs specifically at 10 °C in WT, we observed similar time of day specific transcriptional changes (list provided in Supplementary Dataset S7). Reports that time of the day can modulate the magnitude of the transcriptional response to cold stress have been previously characterized^43,44,67^. In our analysis, we also observed that the upregulation of *CBF2 (AT4G25470)* in response to moderate cold stress (10 °C) is greater at ZT6 (LFC 5.1; FDR 3.1^−03^) vs ZT1 (LFC 4.3; 3.4^−07^).

We identified 13 *HSFs* that are differentially regulated by heat stress and six of them (*HSFA1D*, *HSFA1E*, *HSFA3*, *HSFA6B*, *HSFA7A*, and *HSF4*) showed modulation of the transcriptional response at ZT1 vs ZT6. For example, induction of *HSFA3* in response to heat stress is greater at ZT6 relative to ZT1 (LFC of 3.04 vs 1.85 and FDR of 3.0^−03^ vs 1.35^−05^, respectively) in WT, suggesting that HSFA3 is more sensitive to the heat stress in the early afternoon. Interestingly, the induction of *HSFA3* is also strongly enhanced in the *cca1lhy* (LFC 3.12; FDR 2.11^−08^) and *prr7prr9* (LFC 3.63; FDR 0.001) mutants at 37 °C relative to 22 °C, suggesting that the clock might also play an important role in modulating the transcriptional response of *HSFA3* to high temperature stress during the day.

### The clock controls the magnitude of the transcriptional response for a subset of DEGs

In *Arabidopsis*, up to 50% of the genes that are responsive to heat, cold, and other abiotic stresses show circadian rhythmicity^31–33,68–70^. Our analysis was performed to determine the contribution and connection between time of day and the clock modulation of heat stress responses through identification of the genes underlying this regulation.

Overall, from the thousands of genes that were differentially regulated in our analysis either by time, treatment, or genotype, we obtained 213 genes that were differentially expressed at 10 °C or 37 °C relative to 22 °C in WT, *cca1lhy* or *prr7prr9*, and that were also differentially expressed between WT and clock mutants at similar temperature stresses. Thus, these genes responded to heat (150 genes), cold (55 genes), or both (8 genes) stresses, and are also regulated by the clock (Supplementary Dataset S8). As mentioned earlier, the *cca1lhy* and *prr7prr9* double mutants have compromised circadian phasing, so it is possible that the observed differential response to temperature stress for many of the 213 genes are a result of phase changes and/or indirect feedback regulation^21,63,64^.

**Figure 4:**
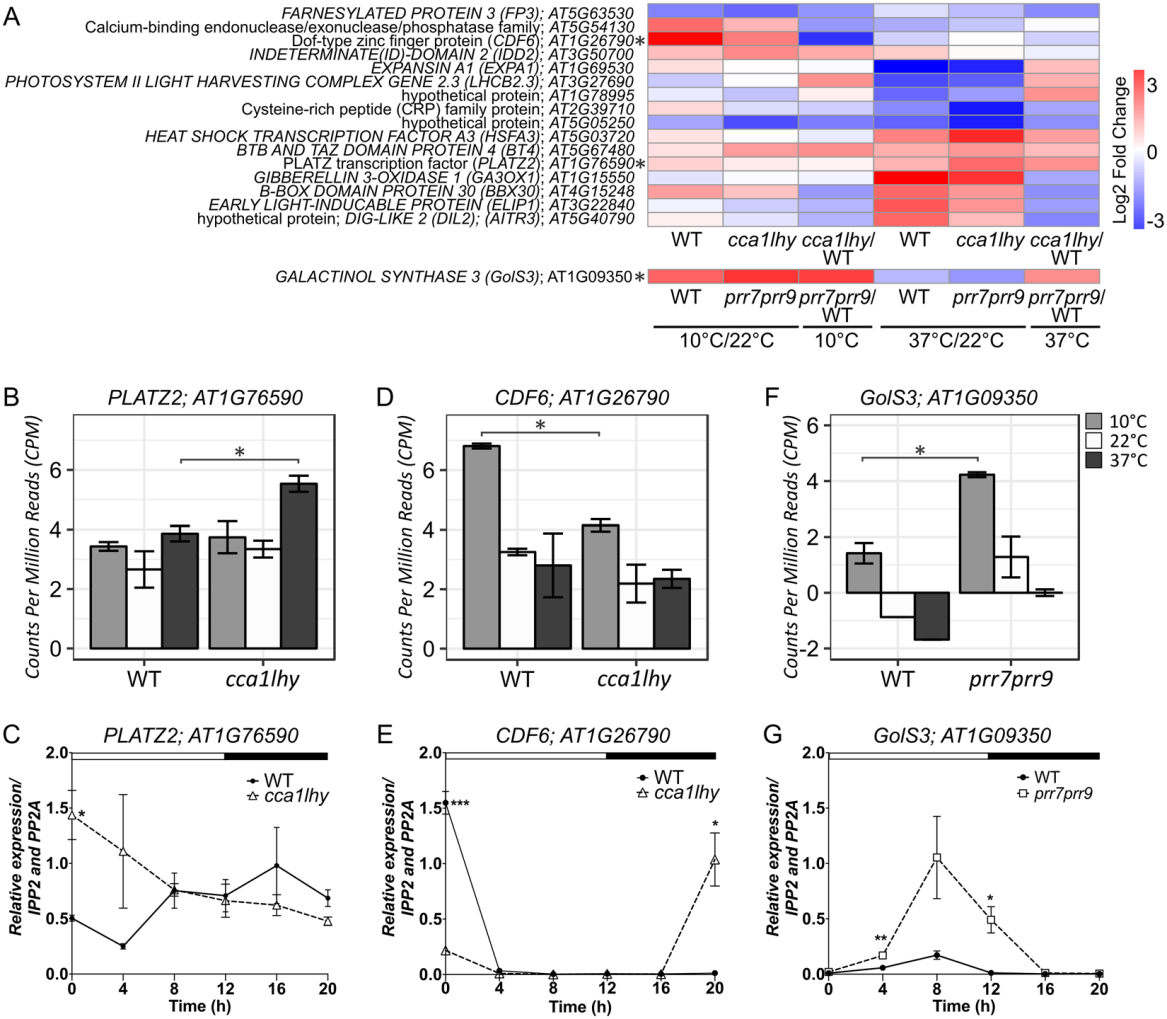
Alteration of gene expression for select clock controlled targets. (**A**) Heatmap of the 17 DEGs that are *mis*-expressed in either *cca1lhy* or *prr7prr9* or relative to WT and are direct ChIP-seq targets of CCA1, LHY, PRR7, or PRR9. Asterisk indicates the 3 genes described in the panels below. (**B**,**D**,**F**) Normalized counts per million (CPM) for *AT1G26790 (CDF6)*, *AT1G76590 (PLATZ2)*, and *AT1G09350 (GolS3)* at 10 °C, 22 °C, or 37 °C. Replicates were averaged and error bars calculated by standard deviation. Significance was determined by LFC > |1| and FDR < 0.05. (**C**,**E**,**G**) qRT-PCR of *AT1G26790*, *AT1G76590*, and *AT1G09350* transcript levels in WT, *cca1lhy*, and *prr7prr9* plants grown in LD cycles for 12 d. mRNA levels were normalized to *IPP2* and *PP2A* expression (mean values ± SD, n = 3, three independent experiments). ***P ≤ 0.0005; **P ≤ 0.005; *P ≤ 0.05, unpaired student t-test.

Therefore, to systematically determine the contribution of the clock mutation in response to heat or moderate cold stress, we filtered the 213 DEGs mentioned above by only considering DEGs having (i) a similar temperature response in both WT and clock mutant *(i*.*e* up-regulated in response to 37 °C compared to 22 °C in both WT and mutant for example), (ii) a differential expression between the WT and clock mutant at the specific stress temperature, but (iii) no differential expression between the WT and clock mutant at normal growth temperature (22 °C). This led to the identification of 69 DEGs for which the magnitude of the transcriptional response to temperature stress is modulated by the clock (Supplementary Dataset S8). These DEGs included genes that are either known to be clock controlled and/or abiotic stress regulated such as *EARLY LIGHT-INDUCABLE PROTEIN (ELIP1; AT3G22840)* and *PHOTOSYSTEM II LIGHT HARVESTING COMPLEX GENE 2*.*3 (LHCB2*.*3; AT3G27690)* (Supplementary Dataset S8). To determine if these 69 DEGs are direct clock target genes, we compared this list of genes with published CCA1, LHY, PRR7, and PRR9 Chromatin Immunoprecipitation followed by deep sequencing (ChIP-seq) (Supplementary Dataset S8)^71–75^. Approximately 25% (17 of 69) of these DEGs were found to be directs targets of one or more clock genes (Fig. 4A; Supplementary Fig. S3 and Dataset S8).

Although several of the 17 direct clock targets exhibit circadian oscillations based on the DIURNAL project, a direct link between the clock and their response to temperature stress was previously unknown^60^. For example, the plant specific *PLATZ TF (PLATZ2; AT1G76590)*, has been reported to have roles in abiotic stress responses such as desiccation tolerance in *Arabidopsis*^76^. In our dataset, *PLATZ2* is significantly upregulated only at 37 °C in *cca1lhy* (LFC 2.2; FDR 1.3^E-05^) relative to WT (LFC 1.2; FDR 4.1^E-03^; Fig. 4B and Supplementary Dataset S8). In addition, *PLATZ2* expression level is altered in the *cca1lhy* mutant significantly at ZT0, which coincides with the peak expression of *CCA1* and *LHY* though expression changes are also observed at other time-points throughout the day, including ZT8, the timing of peak expression for *PLATZ2* (Fig. 4C and Supplementary Fig. S3)^60^. These results suggest that the expression of *PLATZ2* might be negatively regulated by the clock, and the occurrence of the temperature stress response is also directly controlled by the clock.

CCA1 and LHY modulation of transcript abundance in response to cold stress is also observed for *CYCLING DOF FACTOR 6 (CDF6; AT1G26790)*, a member of the *Dof* subfamily, involved almost exclusively in photoperiod flowering and abiotic stress responses in *Arabidopsis* (Fig. 4D)^77,78^. Similar to other members of the family (*CDF1*, *CDF2*, *CDF3* and *CDF5*), *CDF6* shows peak expression in the early morning (Fig. 4E)^77,78^. Some of these *CDFs* are also regulated in the morning by CCA1 and LHY and in the afternoon by PRR9, PRR7, and PRR5^79,80^. Consistent with this observation, *CDF6* showed significantly reduced expression in the *cca1lhy* mutant (Fig. 4D,E). Because CCA1 and LHY are suggested to play a positive role on components of the morning loop such as *PRR7* and *PRR9*, the reduced expression of *CDF6* in the *cca1lhy* mutants might be due to the direct regulation by CCA1 and/or LHY, since *CDF6* is reported to be a target of LHY^21,75^. Alternatively, the reduced expression of *CDF6* in the *cca1lhy* mutant might be due to indirect feedback regulation given that *CDF6* is also a target of PRR9^73^. Interestingly, the significant induction of *CDF6* at 10 °C is reduced almost two-fold in the *cca1lhy* mutant, emphasizing the important role of CCA1 and LHY in the cold response of *CDFs* (Fig. 4D,E).

For the single DEG that was *mis*-regulated in *prr7prr9/WT* comparison based on our stringent selection criteria, *GALACTINOL SYNTHASE 3 (GolS3; AT1G09350)*, we observed up-regulation in *prr7prr9* relative to WT at 10 °C and likely 37 °C (Fig. 4F). *GolS3* belongs to a gene family that has been implicated in drought stress, heat stress, cold stress and oxidative damage^81^. However, very little is known about the precise function of *GolS3*, and the importance of the clock and cold temperature.

Of note, comparison between the time of day heat stress responsive DEGs mentioned in Fig. 3 and the ChIP-seq datasets, revealed that an additional ~11% (166 genes) were targets of CCA1, LHY, PRR7, or PRR9, 5 of which are shared with the 69 DEGs mentioned above, suggesting that both time of day and the clock modulate their transcriptional response to high temperature stress (Supplementary Dataset S8). For example, differential transcriptional response to heat stress for *HSFA3* described in reference to Fig. 2A above, appears to be modulated by both time of day and the clock (Fig. 4A).

It is possible that the DEGs that were not identified as direct clock targets are indirectly regulated by the clock. In addition, although we were able to make direct connections between the clock and a subset of our DEGs, the ChIP-seq experiments were performed at normal growth temperature for *Arabidopsis* (~22 °C). CCA1 has been shown to bind more strongly to *PRR9* at 27 °C compared to 12 °C, and temperature has been implicated in the regulation of alternative splicing for some clock genes suggesting that both differential binding of clock genes and post-transcriptional regulation might contribute to modulating the gene expression of stress responsive genes^82,83^. Therefore, ChIP-seq analysis of CCA1, LHY, PRR7, and PRR9 conducted under similar temperature stress conditions used in this study will likely contribute to defining the regulatory relationship between the clock and temperature-responsive genes. In fact, ChIP-seq analysis of evening expressed clock genes (*LUX*, *ELF3*, and *ELF4*) found that association of these components to target gene promoters was either decreased by high ambient (27 °C) or increased at low (17 °C) ambient temperatures relative to 22 °C, enabling the identification of additional targets that would have otherwise been overlooked^84,85^.

### Clock-controlled and heat stress regulated DEGs reveals specific network connections

Overall from the DEGs including the time of specific and clock controlled DEGs obtained in this analysis, 9% are annotated as TFs. From the genes targeted by CCA1, LHY, PRR7, or PRR9, we selected TFs that showed time of day specific differential changes in response to heat and those whose magnitude of the temperature response was controlled by the clock in our analyses. Of these 74 TFs, six are from the 69 clock-controlled DEGs and one belongs to the 119 time of day specific DEGs described above. We examined the Arabidopsis Cistrome Atlas which contains TFs and their target genes obtained by DNA affinity purification sequencing (DAP-seq)^86^, and found targets for one of these seven DEGs, *HB21*, *HOMEOBOX PROTEIN 21 (AT2G18550)*, a class I *Homeodomain leucine zipper (HD-ZIP)* TF^87^. *HB21* has been shown to be involved in hormone responses and plant development in light limiting conditions^87^.

**Figure 5:**
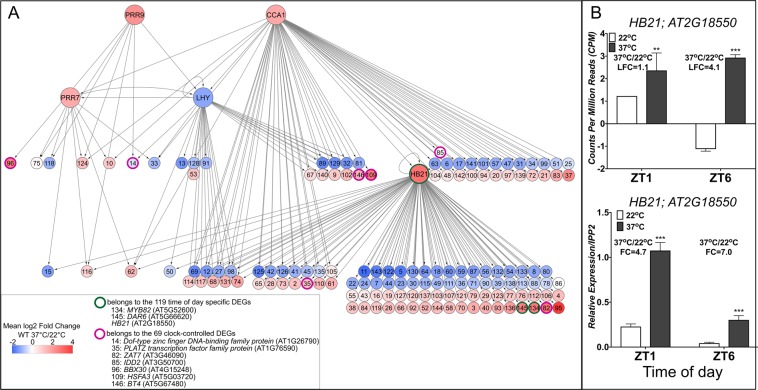
Interaction network of differentially expressed transcription factors. (**A**) Listed in the first level of the network are the 46 specific TFs including *HB21* that are direct targets of CCA1, LHY, PRR7, and/or PRR9 based on published ChIP-seq datasets^71–73^. Second level indicates direct connections for the 97 HB21 targeted DEGs from our dataset, obtained from DNA affinity purification sequencing (DAP-seq) data^86^. The 28 TFs (DEGs) shared between CCA1, LHY, PRR7, PRR9, and HB21 are indicated. Arrows in the network represents an interaction between the TF and its target gene based on either Chromatin Immunoprecipitation followed by deep sequencing (ChIP-seq) or DAP-seq. TFs circled in pink are from the list of clock controlled DEGs, and in green are genes from the time of day regulated DEGs. Upregulated and down regulated DEGs in response to 37 °C compared to 22 °C are indicated by shades of red or blue depending on the LFC values (> |1| and FDR < 0.05), respectively. Identity of the genes represented by numbers in each connection are listed in Supplementary Dataset S9. Network was visualized using Cytoscape software version 3.3.0^93^. (**B**) Time of day expression of *HB21* based on RNA-seq and qRT-PCR analysis. For RNA-seq, data are based on Normalized Counts Per Million Reads (CPM), and LFC was calculated based on 37 °C/22 °C at either ZT1 or ZT6. qRT-PCR was performed as described in Fig. 1 and normalized to *IPP2* expression, and Fold Change (FC) was calculated based on expression values at 37 °C relative to 22 °C. ***P ≤ 0.0005 and **P ≤ 0.005, unpaired student t-test.

We generated a gene regulatory network composed of 148 TFs including CCA1, LHY, PRR7, PRR9, and HB21 and their respective TF targets that responded to heat in a time of day and/or in a clock dependent context, and revealed 208 total connections (Fig. 5A and Supplementary Dataset S9). At all levels (clock regulated or *HB21* regulated DEGs), ~50% of the target genes were either up- or down-regulated in response to heat, and based on GO function analysis are enriched for leaf senescence, response to hormones, and response to light stimulus, consistent with the reported function of *HB21* (Fig. 5A). In this hierarchical network, *HB21* targets 97 TFs including 70 that are not targeted by the clock components. Interestingly, the transcriptional response of *HB21* to heat stress is modulated by time of day and showed a significant fold increase in expression at ZT6 in our RNA-seq analysis and confirmed by qRT-PCR (Fig. 5B). In addition, a previously conducted ChIP-seq analysis revealed *HB21* as a direct target of CCA1, although in our data, *HB21* is not significantly upregulated in response to heat stress (37 °C vs 22 °C) in the *cca1lhy* or *prr7prr9* mutant or compared to WT (Supplementary Dataset S1 and S2)^71^. Interestingly, 28 HB21 target genes are also targets of CCA1, LHY, PRR7 or PRR9, such as *PLATZ2*, whose response to heat stress is modulated by the clock (Figs 4B,C and 5A). To the best of our knowledge, this is the first report linking HB21 and ~100 of its target genes to heat stress, time of day specific expression, and the clock in *Arabidopsis*. This integration of multiple data sources allowed us to build a hierarchical regulatory network that highlights the contribution of the dimension of time to specific environmental responses and growth and development. Future work to define the underlying mechanisms of this interaction along with integration of other stressomes will significantly contribute to our understanding on how plants interact with a changing environment.

## Conclusion

One of the mechanisms used by plants to respond to environmental stresses is coordinated through an elaborate gene regulatory network that involves the circadian clock. For heat stress responses, understanding how time of day and the circadian clock alters the transcriptional response such as the occurrence and magnitude of the response is essential. In this study, we performed a transcriptomic analysis to assess the extent of the time dimension and the contribution of the clock on the transcriptional dynamics of temperature stress responses during the day when plants should be primed in anticipation of increasing temperatures. Our analysis revealed that although a large subset of genes is induced by heat stress during the early morning and early afternoon, the occurrence or magnitude of this transcriptional induction depends on the time of day and/or the circadian clock. The precise regulatory mechanisms underlying this clock environment interaction in terms of heat stress remains to be determined. Our data reflects responses limited to 1 h and two time-points, therefore future work to detect heat stress responses that accumulate after prolonged exposure or different times of the day will further provide insights to the contribution of time of day on temperature stress responses. DEGs identified in this study should help to guide mechanistic studies, and integrate intricate gene regulatory networks together with the massive amounts of publicly available transcriptomic data underlying the abiotic stress response pathways.

## Experimental Procedures

### Plant materials and growth conditions

In all experiments, *Arabidopsis thaliana* Columbia-0 (Col-0) was used as wild-type (WT). The *cca1-1/lhy-20 (cca1lhy)* was generated by crossing the previously characterized *cca1-1* mutation in Col-0 with *lhy-20* also in Col-0 background^28,59^. The *prr7-3/prr9-1 (prr7prr9)* in Col-0 was previously characterized^21,58^. Seeds were surface sterilized and stratified in the dark at 4 °C for 3 days. Plants were grown on plates containing Murashige and Skoog (MS) medium supplemented with 1.5% sucrose (wt/vol) in 12 h light and 12 h dark (LD) cycles for 12 days at constant 22 °C and 90 µm light intensity. For the RNA-sequencing experiment, a subset of plants was transferred for 1 h to a growth chamber set to either 10 °C or 37 °C on day 13. For *cca1lhy*, plants were transferred at lights on (ZT0) and for *prr7prr9*, 5 h after lights on. Whole seedlings were collected one hour after temperature treatments corresponding to ZT1 and ZT6.

### RNA extraction and RNA-sequencing library preparation

Total RNA was extracted using the RNeasy Plant Mini Kit (Qiagen, Hilden, Germany) following manufacturer’s protocol. The extracted RNA was DNaseI treated to remove contaminating DNA (Millipore Sigma, Burlington, MA, USA). Next between 2 µg and 5 µg of total RNA was mRNA purified with Dynabeads Oligo-dT(25)^88^. Libraries were prepared as described previously^89^. Modifications to the protocol are as follows: for the adapter ligation, EDTA was not added to the samples during adapter ligation, and samples were always eluted from the beads using RNAse-free water instead of 10 mM Tris-HCl pH 8. In the final enrichment, Kapa HIFI (Kapa Biosystems, Wilmington, MA, USA) was used instead of Phusion Polymerase, and we performed 15 cycles to amplify the libraries. Final libraries were either purified by Ampure XP beads or SDS-PAGE gel extraction^90,91^. Library concentration and quality were verified using a Qubit 2.0 Fluorescence Reader (ThermoFisher Scientific) and Bioanalyzer 2100 (Agilent Genomics). Libraries consisting of four biological replicates for Col-0 and *cca1lhy* after one hour treatment (ZT1), and two replicates of Col-0 and *prr7prr9* after one hour treatment (ZT6) for each temperature were multiplexed with unique barcodes and sequenced.

### RNA-sequencing and data analysis

Single-end 75 base pair sequences were generated for each mRNA library using the NextSeq500 (Illumina) at the UC Riverside (UCR) Institute for Integrated Genome Biology (IIGB) Genomics Core facility. Data analysis was conducted by following the systemPipeR workflow using a computer cluster operated by the UCR IIGB facility, specifically using R version 3.4.3^92^. To control quality of reads, we used cutadapt with default settings and FastQC reports^92^. Trimmed reads were mapped to the TAIR10 genome using the alignment software, TopHat2 (2.0.14) and Bowtie2 (2.2.5)^92^. Exons were counted by “union” mode with GenomicFeatures using the Araport11 gff (201606). An offset of 5 counts was applied to all counts followed by library scaling by edgeR.calcNormFactors^92^. Limma was employed to conduct differential gene expression analysis specifically using quantile normalization with voom^92^. Log-fold change (LFC) was calculated by subtracting the normalized CPM values between treatments, while false discovery rate (FDR) was calculated with the Benjamini & Hochberg method^92^. Differentially expressed genes (DEGs), used for downstream analysis and heatmap clustering, were selected by filtering for Log_2_ Fold Change (LFC) > |1| and FDR < 0.05. Clustering for heatmaps utilized the Euclidean method with partition around medoids^92^.

### Comparison with other temperature stress experiments

Genevestigator was used to identify heat and cold stress experiments performed in Col-0 plants^46^. The Genevestigator accession numbers used were: AT-00120, AT-00176, AT-00221, AT-00230, AT-00288, AT-00389, AT-00402, AT-00500, AT-00633, AT-00640, AT-00641, AT-00645, AT-00654, AT-00670, and AT-00751.

### Quantitative real-time PCR

Seeds were prepared as described above and grown in LD conditions at 22 °C for 12 d. Samples were collected every 4 h in LD and total RNA was isolated with the Qiagen RNeasy plant mini kit (Qiagen, Hilden, Germany). cDNA was synthesized using 1 µg of total RNA and was reverse-transcribed with the iScript cDNA synthesis kit (Bio-Rad). See Supplementary Table S1 for gene specific primers and qRT-PCR conditions used here.

### Interaction network analysis

To analyze interactions between selected TFs, published Chromatin Immunoprecipitation followed by deep sequencing (ChIP-seq) data for *CCA1*, *LHY*, *PRR7* and *PRR9*, and DNA Affinity Purification sequencing (DAP-seq) data for *HB21* were used^71–73,86^. *CCA1*, *LHY*, *PRR7*, *PRR9*, and *HB21* were defined as sources. Targets were restricted to differentially expressed transcription factors. Interaction networks were visualized using Cytoscape software version 3.3.0^93^.

## Supplementary information

Supplementary Data

Dataset S1

Dataset S2

Dataset S3

Dataset S4

Dataset S5

Dataset S6

Dataset S7

Dataset S8

Dataset S9

## Data Availability

The sequences reported in this paper have been deposited in the National Center for Biotechnology Information Gene Expression Omnibus (GEO) database and can be accessed through GEO Series accession GSE116004.
