# Supplementary material for: Contribution of time of day and the circadian clock to the heat stress responsive transcriptome in Arabidopsis
Source: Sci Rep. 2019 Mar 18;9:4814. doi: 10.1038/s41598-019-41234-w (PMC6423321; doi:10.1038/s41598-019-41234-w)
Supplement: Supplementary file 1 — Supplementary Data [file 41598_2019_41234_MOESM1_ESM.pdf]

Supplementary Figures and Table - SREP-18-43668

Contribution of time of day and the circadian clock to the heat stress responsive transcriptome in *Arabidopsis*

Authors

Emily J Blair[1], Titouan Bonnot[1], Maureen Hummel[1], Erika Hay[1], Jill M Marzolino[1] and Ivan A Quijada[1], and Dawn H Nagel[1*]

[1] Department of Botany and Plant Sciences, University of California, Riverside, Riverside, California

[*] Corresponding author

Dawn H Nagel

dawnn@ucr.edu

Tel: 951-827-4425

Supplementary Figures and Table:

Supplementary Fig. S1: Comparison between our dataset and published datasets.

Supplementary Fig. S2: Comparison of the number of up and down-regulated genes at different temperatures.

Supplementary Fig. S3: Isolation of direct clock targets and the expression profile of the respective clock genes.

Supplementary Table S1: PCR conditions and primer sequences used in qRT-PCR

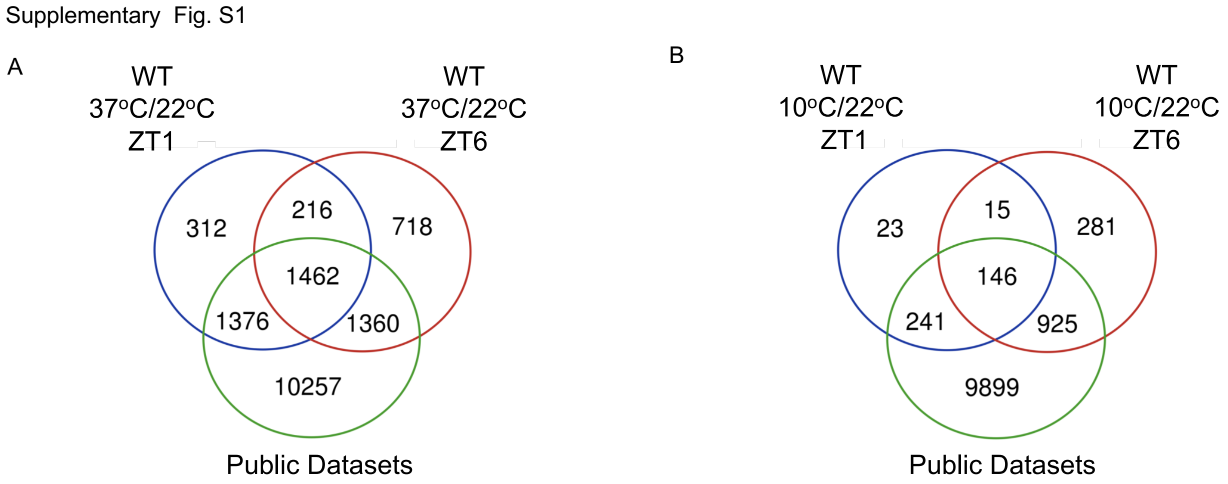

Supplementary Fig. S1: Comparison between our dataset and published datasets. (A and B) Venn diagrams displaying overlapping DEGs between heat (A) and cold (B) stress in WT at ZT1 and ZT6 compared to published datasets available on Genevestigator, and most recent heat and cold stress RNA-seq datasets at the time of this analysis [39,43,54]. For the Calixto *et al.,* 2018 dataset, we used DEGs from timepoints similar to the time of day (ZT0 and ZT6) for our analysis. For the Albihlal *et al.,* 2018, we used RNA-seq from the WT heat stressed, WT non-stressed (NS), and the HSFA1b OX NS experiments.

Supplementary Fig. S2

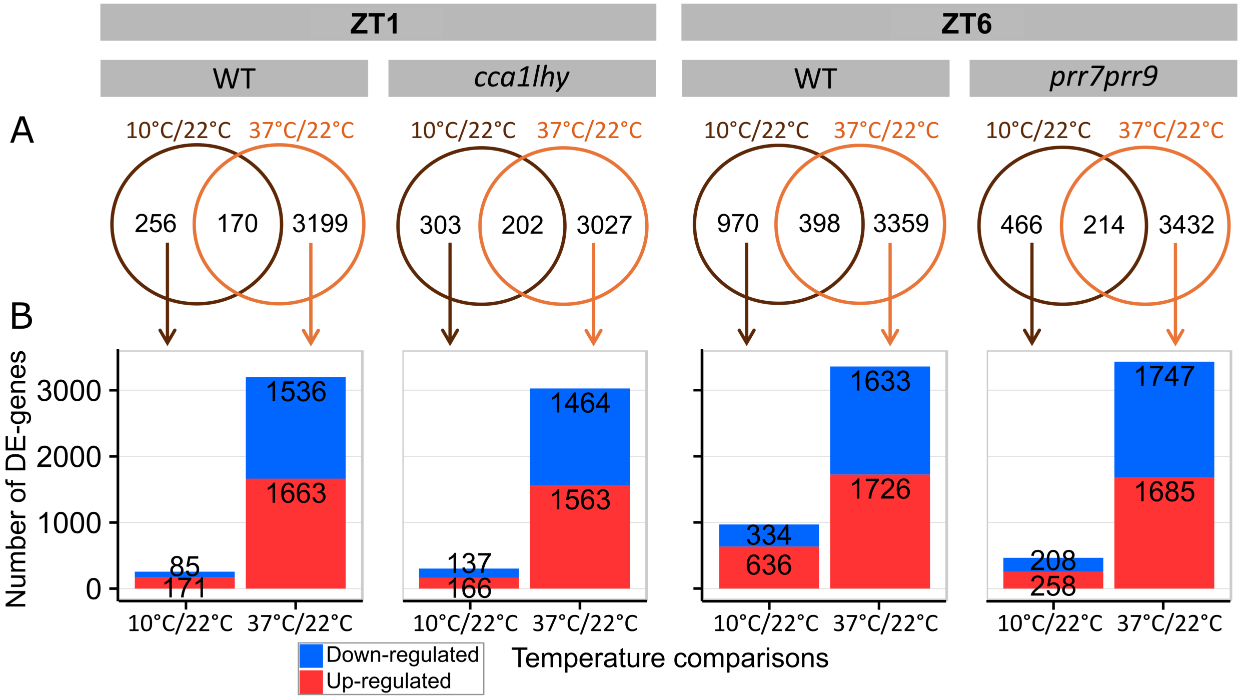

Supplementary Fig. S2: Comparison of the number of up and down-regulated genes at different temperatures. (A) Venn diagrams display DEGs that overlap between temperature treatments in WT or *cca1lhy* at ZT1 and WT or *prr7prr9* at ZT6 after lights ON. (B) Number of differentially expressed genes comparing temperature treatments by genotype and time of day.

Supplementary Fig. S3

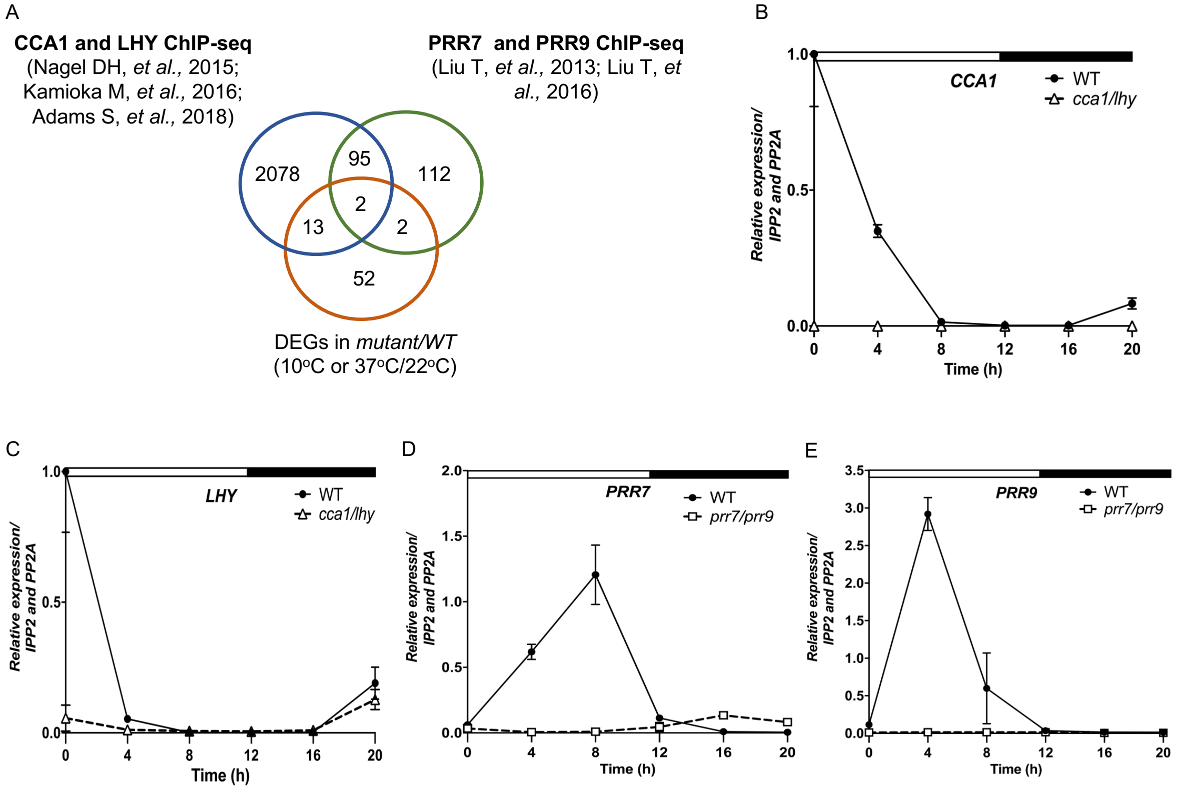

Supplementary Fig. S3: Isolation of direct clock targets and the expression profile of the respective clock genes. (A) Comparison between CCA1, LHY, PRR7, and PRR9 direct targets and differentially regulated genes (DEGs) in *cca1lhy*/WT and *prr7prr9*/WT. Venn diagram showing the overlap between the high confidence CCA1 targets in LD (Nagel *et al.*, 2015), non-overlapping CCA1 targets from Kamioka *et al.*, 2016, LHY targets (Adams *et al.*, 2018), PRR7 targets (Liu *et al.* 2013), and PRR9 targets (Liu *et al.* 2016), and DEGs for *mutants* compared to WT [69–73]. (B-E) Relative expression of *CCA1, LHY, PRR7,* and *PRR9*. qRT-PCR of *CCA1, LHY, PRR7*, and *PRR9* transcript levels in WT, *cca1lhy*, and *prr7prr9* plants grown in LD after 10 d entrainment in 12 h:12 h LD cycles with collection on 12 d. mRNA levels were normalized to *IPP2* and *PP2A* expression (mean values ± SD, n = 3, three independent experiments). X-axis, time in hours (h) and Y-axis, relative expression.

Supplementary Table 1: PCR Conditions and Primer Sequences used in qRT-PCR

PCR conditions were 95 °C for 3 min followed by 40 cycles at 95 °C for 10 s, 55 °C for 15 s, and 72 °C for 15 s. *Protein Phosphatase 2A (PP2A) and isopentenyl-diphosphate delta-isomerase II (IPP2)* were used as normalization controls.

PRR9-F: 5'-GCCAGAGAGAAGCTGCATTGA-3'
PRR9-R: 5'-CCTGCTCTGGTACCGAACCTT-3'
LHY-F: 5'-CAACAGCAACAACAATGCAACTAC-3'
LHY-R: 5'-AGAGAGCCTGAAACGCTATACG-3'
PRR7-F: 5'-CTTTCTCAAGGTATAATCCAGCC-3'
PRR7-R: 5'-ACAATCATATGCTGCTTCAGTC-3'
CCA1-F: 5'-CAGCTCCAATATAACCGATCCAT-3'
CCA1-R: 5'-CAATTCGACCCTCGTCAGACA-3'
GolS3-AT1G09350-F: 5'-ACAGGCCAAGAAGGAAATATGG-3'
GolS3-AT1G09350-R: 5'-GATGGAGCTTTGGCACATTG-3'
PLATZ2-AT1G76590-F: 5'-TGGCTAATCCCAATGCTAAGAG-3'
PLATZ2-AT1G76590-R: 5'-ACATGTTGCACTCGCTTTTG-3'
CDF6-AT1G26790-F: 5'-GACTTGTATTGTCAGTAACAGATTGG-3'
CDF6-AT1G26790-R: 5'-TGGCTGGACAATTACACCG-3'
HB21-AT2G18550-F: 5'-CTTCTACTCATTTCTCAATTGTACCC-3'
HB21-AT2G18550-R: 5'- CACCCATTGCCTTCGTTTTC-3'
IPP2-F: 5'- CTCCCTTGGGACGTATGCTG-3'
IPP2-R: 5'- TTGAACCTTCACGTCTCGCA-3'
PP2A-F: 5'-TAACGTGGCCAAAATGATGC-3'
PP2A-F: 5'-GTTCTCCACAACCGATTGGT-3'
